# Increased IL-17, a Pathogenic Link between Hepatosplenic Schistosomiasis and Amyotrophic Lateral Sclerosis: A Hypothesis

**DOI:** 10.1155/2014/804761

**Published:** 2014-07-23

**Authors:** Oswald Moling, Alfonsina Di Summa, Loredana Capone, Josef Stuefer, Andrea Piccin, Alessandra Porzia, Antonella Capozzi, Maurizio Sorice, Raffaella Binazzi, Lathá Gandini, Giovanni Rimenti, Peter Mian

**Affiliations:** ^1^Division of Infectious Diseases, Ospedale Generale, 39100 Bolzano, Italy; ^2^Division of Neurology, Ospedale Generale, 39100 Bolzano, Italy; ^3^Radiology, Ospedale Generale, 39100 Bolzano, Italy; ^4^Division of Hematology, Ospedale Generale, 39100 Bolzano, Italy; ^5^Department of Experimental Medicine, Sapienza University, 00161 Rome, Italy

## Abstract

The immune system protects the organism from foreign invaders and foreign substances and is involved in physiological functions that range from tissue repair to neurocognition. However, an excessive or dysregulated immune response can cause immunopathology and disease. A 39-year-old man was affected by severe hepatosplenic schistosomiasis *mansoni* and by amyotrophic lateral sclerosis. One question that arose was, whether there was a relation between the parasitic and the neurodegenerative disease. IL-17, a proinflammatory cytokine, is produced mainly by T helper-17 CD4 cells, a recently discovered new lineage of effector CD4 T cells. Experimental mouse models of schistosomiasis have shown that IL-17 is a key player in the immunopathology of schistosomiasis. There are also reports that suggest that IL-17 might have an important role in the pathogenesis of amyotrophic lateral sclerosis. It is hypothesized that the factors that might have led to increased IL-17 in the hepatosplenic schistosomiasis *mansoni* might also have contributed to the development of amyotrophic lateral sclerosis in the described patient. A multitude of environmental factors, including infections, xenobiotic substances, intestinal microbiota, and vitamin D deficiency, that are able to induce a proinflammatory immune response polarization, might favor the development of amyotrophic lateral sclerosis in predisposed individuals.

## 1. Introduction

Schistosomiasis is a major tropical parasitic disease caused by blood-dwelling fluke worms of the genus* Schistosoma*. It is contracted by humans when wading in bodies of water contaminated with the free-swimming cercariae, the larval and infective form of the schistosomes, released from aquatic vector snails. Cercariae penetrate the skin, reach the blood circulation, and mature into adult worms and in the case of* Schistosoma mansoni* home to the mesenteric venous vasculature, where male and female mate and lay 100–300 eggs per day [1, 2]. The lifespan of an adult schistosome averages 3–5 years but can be as long as 30 years [3]. The eggs then exit the vasculature to enter the intestinal lumen and are set free in search of appropriate vector snails. However, many eggs embolize and are trapped mostly in the liver (hepatosplenic schistosomiasis), less frequently in the central nervous system (neuroschistosomiasis), where they precipitate an immune reaction of varying degree. Most individuals develop a relative mild “intestinal” schistosomiasis, whereas 5–10% suffer the severe hepatosplenic form of disease, in which there is progressive liver fibrosis, portal hypertension, splenomegaly, esophageal varicose veins, gastrointestinal hemorrhage, and death [4, 5].

Amyotrophic lateral sclerosis (ALS) is a fatal neurodegenerative disease characterized by progressive and selected loss of both upper and lower motor neurons. Patients experience signs and symptoms of progressive muscle atrophy and weakness and increased fatigue, which typically lead to respiratory failure and death. Median survival is 2–4 years from onset; only 1–10% of patients survive beyond 10 years. Less than 10% of ALS cases are familial with 20% of these cases linked to various mutations in the Cu/Zn superoxide dismutase 1 (SOD1) gene [6, 7]. The etiology of ALS is unknown. A wide range of contributing factors including genetic mutations and polymorphisms, oxidative stress, and neuroinflammation have been identified; however the initiating or most relevant ones are unknown [6–12]. There were more than 2000 publications on ALS only in 2013. This growing knowledge will hopefully move towards the discovery of an effective treatment and prevention of this tragic disease. We report on a 39-year-old man who presented with progressive hemiparesis of unknown origin, lupus-like autoimmune phenomena, and a suspected reactivation of* M. tuberculosis* infection. He was subsequently diagnosed as suffering from severe hepatosplenic schistosomiasis* mansoni* and amyotrophic lateral sclerosis. In an attempt to understand the diseases recent advances in immunology were reviewed and it may be that common pathogenic mechanisms might underlie the different diseases.

## 2. Case Report

A 39-year-old man from Ghana, who had been resident in Italy for five years, was admitted to hospital in April 2013 because of slowly progressive gait disturbances and right hemiparesis which had developed within the last two months. His past medical history was unremarkable. No cerebral vascular lesions were seen neither on magnetic resonance angiography or Doppler sonography. Instead, liver cirrhosis with portal hypertension, esophageal varicose veins, and splenomegaly was detected. He was a mild occasional alcohol drinker and viral hepatitis screening was negative (for laboratory values see Table 1). Hematologic malignancy and leishmaniasis were excluded by bone marrow examination. The presence of antinuclear antibodies, anticardiolipin antibodies detected by TLC immunostaining [13], and lupus anticoagulant, the decrease of the complement factors C3 and C4 were reminiscent of systemic lupus erythematosus (Table 1). Findings of apical scars and calcification of hilar lymph nodes of the right lung detected by computer tomography (CT) and a 15 mm cutaneous tuberculin reaction indicated a* Mycobacterium tuberculosis* infection. Therefore standard antituberculous therapy was initiated, considered also as chemoprophylaxis in the perspective of future corticosteroid treatment. 25-hydroxy-vitamin D 10,000 IU (250 *μ*g) weekly was added to treatment. Sufficient criteria for the diagnosis of systemic lupus erythematosus (SLE) would had been fulfilled [14]; however, such extreme liver and spleen changes as in this patient are not usually seen in SLE.

**Figure 1:**
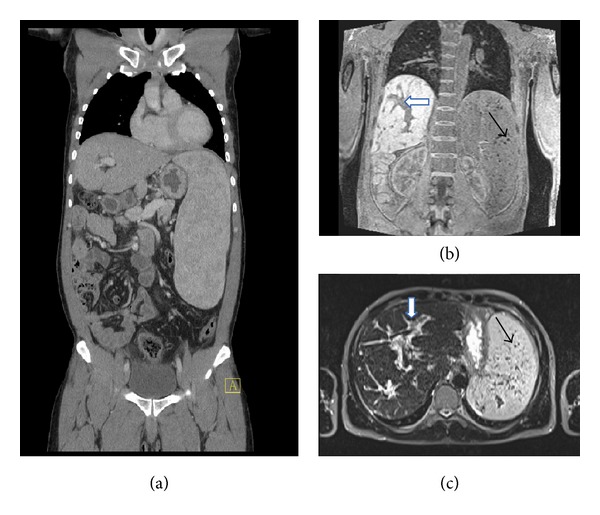
(a) Computerized tomography (CT) imaging showing the enlarged spleen (b) and (c) magnetic resonance imaging (MRI) demonstrating the periportal fibrosis (white arrows) and the Gamna-Gandy bodies (siderotic nodules) in the spleen (black arrows).

Indeed, a high titer of anti-*Schistosoma* antibodies was demonstrated. Retrospectively, the ultrasound-, CT-, and MR-imaging that showed the extensive periportal fibrosis of the liver and the huge spleen with a diameter of 24 cm and unusual spots (Figure 1) turned out to be characteristic for hepatosplenic schistosomiasis* mansoni*. Seven years earlier in the district of Dunkwa in Offin, Ghana, the patient had to wade through water for about 10 minutes daily for more than one year while going to work to cut large trees and requiring physical exertion. Praziquantel 60 mg/kg daily for three days, methyl-prednisolone 1 g daily for five days, and then prednisone 1 mg/kg, tapered in the next three months, were given [15]. Subsequently an improvement in the strength of the right arm was observed for only a few weeks, followed by a worsening of the movement disorder.

Physical examination four months following his first hospitalization revealed muscle weakness also on the left side. There was increased muscle rigidity, hypotrophic thenar muscles of the right hand, hyperreflexia, and spontaneous and triggered muscle fasciculations. Spirometry showed evidenced deficits of the inspiratory and exspiratory musculature. The patient did not report neither sphincter dysfunction nor pain or sensitivity alterations. This pattern was indicative of motor neuron disease and of amyotrophic lateral sclerosis, as were the electromyographic findings. Repeated MRI showed mild diffuse signal hyperintensities of the white matter in the centrum semiovale regions by FLAIR imaging. The gadolinium enhancement of the meninges at the vertebral level D4-D6 was compatible with a reaction to embolized ova of schistosome, but these alterations did not explain the neurological symptoms. Because praziquantel had been given without considering the interaction with rifampin [16], rifampin was stopped. One month later praziquantel was repeated at a dose of 80 mg/kg daily for three days considering the interaction with prednisone [17] at that time given at a dose of 25 mg daily. The movement disorder did not improve despite daily physiotherapy exercises. In the absence of any effective treatment for amyotrophic lateral sclerosis being available to the patient, he returned to Ghana in November 2013.

Thereafter IL-17 has been determined in four serum samples previously stored, which were taken at monthly intervals. IL-17 cytokine was assayed by human-specific ELISA kit (R&D Systems) at the Department of Experimental Medicine, Sapienza University, Rome. The first sample was taken before initiating treatment with praziquantel and high dose corticosteroids. The results were IL-17 672.2 pg/mL, 36.5 pg/mL, <10 pg/mL, and 32.9 pg/mL, respectively, (normal value <31.2 pg/mL).

## 3. Discussion

### 3.1. Diagnostic Considerations

According to the new SLICC classification criteria for systemic lupus erythematosus (SLE) at least 4 out of 17 criteria are necessary for the diagnosis of SLE [14]. Of these 17 classification criteria the described patient satisfied the following 7 criteria: neurologic symptoms, leukopenia, lymphopenia, thrombocytopenia, antinuclear antibodies, antiphospholipid antibodies (anticardiolipin antibodies, lupus anticoagulant), and reduced complement C3, C4. But such extreme hepatosplenomegaly (Figure 1) is usually not seen in SLE. Indeed, the extensive periportal fibrosis, the marked hypertrophy of the left hepatic and caudate lobe, the marked hypotrophy of the right hepatic lobe, and the huge spleen with spots (siderotic nodules, Gamna-Gandy bodies), which was a pattern of abnormalities that had never been seen at the General Hospital of Bolzano, turned out to be characteristic for hepatosplenic schistosomiasis and allowed the distinction of hepatosplenic schistosomiasis from viral or alcohol induced liver cirrhosis [18–20]. Splenic Gamna-Gandy bodies can also be observed in sickle cell anemia [21]. In endemic areas these sonographic findings are used as a noninvasive diagnostic test, replacing invasive liver biopsy [18]. Because of the thrombocytopenia and the altered coagulation tests (Table 1), liver biopsy and lumbar puncture were not carried out in the patient.

The nonspecific mild diffuse white matter hyperintensities seen in the MR-FLAIR-imaging of the patient, can be seen in SLE [22], in neurologically asymptomatic hepatosplenic schistosomiasis* mansoni* [23], and in other cerebral small vessel disease [24], and are not indicative of motor neuron disease. The antiphospholipid antibodies might have caused cerebral microangiopathy and favored blood brain barrier disruption and neuroimflammation [25]. Schistosome eggs can reach the CNS trough retrograde venous flow in the valveless Batson vertebral venous plexus, which connects the portal venous system and the venae cavae to the spinal cord and cerebral veins [2]. The gadolinium enhancement of the meninges at the vertebral level D4-D6 of the patient might have reflected an immunoreaction to schistosome eggs but do not explain the selective motor neuron disease. Spinal neuroschistosomiasis of* Schistosoma mansoni* usually manifests with lumbar pain, lower limb radicular pain, muscle weakness, sensory loss, and bladder dysfunction [2, 14]. The question was whether there was a link between hepatosplenic schistosomiasis and ALS.

### 3.2. Hepatosplenic* Schistosmiasis mansoni* and IL-17

In schistosomiasis the host mounts a pathogenic immune response against tissue-trapped parasite eggs. The CD4 T cell mediated granulomatous inflammation varies greatly in magnitude in humans and among mouse strains in experimental models [5]. Mouse strains which develop severe immunopathology show substantial Th17 as well as Th1 and Th2 cell responses; a solely Th2-polarized response is only observed in low-pathology strains such as the C57BL/6 mice [26]. The ability to mount pathogenic Th17 cell responses depends on the production of IL-23 and IL-1*β* by antigen presenting cells following recognition of egg antigens by pathogen associated molecular patterns (PAMPs) recognition receptors (PRRs) [27]. IL-1*β* contributes to the induction of Th17 cells and IL-23 is necessary for their maintenance and proliferation. Low-pathology C57BL/6 mice immunized subcutaneously with a soluble schistosome egg antigen preparation in complete Freund's adjuvant (CFA), that contains mainly heat killed* M. tuberculosis,* prior to and during infection with schistosomes, were shown to develop severe immunopathology. If in these mice the genes for p40 or p19, the two peptide components of IL-23 were knocked out, they became resistant to the induction of the severe immunopathology. Knocking out IL-12p35, a component of IL-12, did not prevent the severe schistosomiasis induced immunopathology. This indicates that an IL-17 producing T cell population, likely driven by IL-23, significantly contributes to the severe immunopatology in schistosomiasis [28]. T-helper 17 cells have been associated with pathology in* Schistosoma haematobium*-infected children [29].

In other experiments it was demonstrated that schistosome-specific IL-17 induction by dendritic cells from low-pathology C57BL/6 mice is normally inhibited by their induction of IL-10. In vitro, simultaneous stimulation of schistosome-exposed C57BL/6 dendritic cells with a heat-killed bacterium enables these cells to overcome IL-10 inhibition and to induce IL-17. This schistosome specific IL-17 was dependent on IL-6 production by the copulsed dendritic cells [30]. In vivo, coimmunisation of C57BL/6 animals with bacterial and schistosome antigens also resulted in schistosome-specific IL-17 production and this response was enhanced in the absence of IL-10 mediated immune regulation [30]. These experiments suggest that the balance of pro- and anti-inflammatory cytokines that determines the severity of pathology during schistosome infection can be influenced not only by the host and parasite, but also by concurrent bacterial or mycobacterial stimulation. On the other side, coinfection with intestinal nematodes was shown to reduce hepatic egg-induced immunopathology due to damping pathogenic Th17 cell responses by promoting regulatory mechanisms such as those afforded by alternatively activated macrophages and T regulatory cells [31]. It can be concluded that the pathological immune response to schistosome infection, represented by increased IL-17, is affected by multiple factors including intestinal parasite exposure, host variability, bacterial or mycobacterial infection, and commensal microbiota.

### 3.3. Amyotrophic Lateral Sclerosis and IL-17

There is compelling evidence that neuroinflammation is involved in the pathogenesis of ALS [32–34]. But neuroinflammation can exert both neuroprotective and neurodestructive effects, depending on the balance and timing of the different immune responses [32–34]. IL-17 was discovered to be a key player in the pathogenesis of multiple sclerosis (MS). P40 or p19 gene knockout mice are resistant to induction of experimental autoimmune encephalomyelitis, the experimental model of multiple sclerosis [35]. As mentioned above, p40 and p19 are the two peptide components of IL-23, the cytokine necessary for maintenance and proliferation of Th17 cells [35].

Recently, increased levels of IL-17 have been found in the blood and in the CSF of the majority of patients affected by ALS [35–37]. But there are only few reports on IL-17 in ALS. Fiala and colleagues reported that the IL-17 serum level was increased above the highest observed level in control subjects of 40 pg/mL in 65% of 32 patients with ALS and in 4 of 4 patients with autoimmune disease [37]. In the described patient the IL-17 serum level was 672.2 pg/mL in the first blood sample, which was taken before treatment with praziquantel, and high dose corticosteroids were initiated. The normalization of the IL-17 values observed in the following three samples may be a consequence of treatment with high dose corticosteroids, with praziquantel, or a consequence of other unknown reasons. In vitro, mononuclear cells treated with superoxide dismutase 1 (SOD1) aggregates, misfolded SOD1, oxidized SOD1, or with mutant SOD1 produced IL-1*β*, IL-6, and IL-23, the cytokines that induce IL-17 [37, 38]. Stimulation of peripheral blood mononuclear cells by mutant SOD1 induced higher transcripts of IL-1*α* and IL-6, but lower transcripts of IL-10 in mononuclear cells of ALS patients as compared to controls [37]. This suggests that the vulnerability to ALS may be linked to the mode of the immune response. In the transgenic mouse model CNS-targeted production of IL-17 induces activation of astrocytes and microglia, microvascular pathology and enhances the neuroinflammatory response to systemic endotoxemia [39].

### 3.4. IL-17

**Figure 2:**
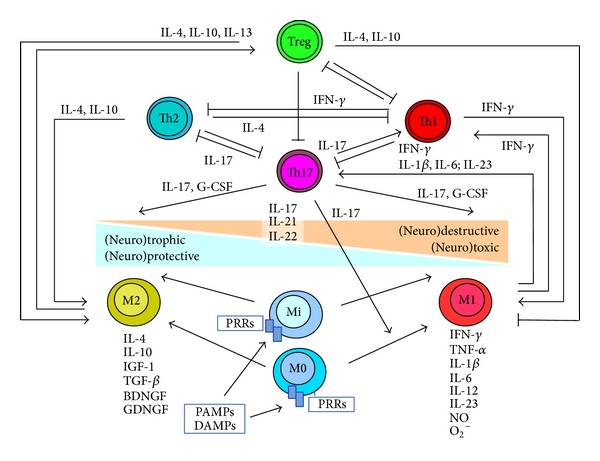
Simplified hypothetical model of immune cell interaction. ↑ = activation, induction; T = inhibition, reduction; BDNF = brain derived neurotrophic factor; DAMP = danger associated molecular pattern; G-CSF = granulocyte-colony stimutating factor; GDGF = glial-cell-derived neurotrophic factor; IGF-1 = insulin-like growth factor; IL = interleukin; Mi = microglia; M0 = nonactivated macrophages; M1 = classically activated macrophages; M2 = alternatively activated macrophages; PAMP = pathogen associated molecular pattern; PRRs = PAMP recognition receptors; and TGF-*β* = transforming growth factor-*β*.

IL-17, produced also by non-Th17 cells, functions as a first line of defense and represents a bridge between innate and adaptive immune response (for immune cell interaction see Figure 2) [40–43]. IL-17 protects the host from bacterial and fungal infections, particularly at the mucosal surfaces. IL-17 has also potent inflammatory potential and was shown to be the “*superstar*” in chronic inflammatory conditions [40–44]. Multiple pathogen associated molecular pattern (PAMP) recognition receptors (PRRs), for example, Toll-like receptors, NOD-like receptors, C-type lectins, on macrophages and other innate immune cells sense pathogen associated molecular patterns (PAMPs), xenobiotic substances, and endogenous “danger” associated molecular patterns (DAMPs) (e.g. aggregated, misfolded, oxidized, or mutant SOD1), and activate the immune response. Disease susceptibility or disease outcome may result from exposure to one or multiple infectious pathogens, xenobiotic substances (e.g. heavy metals), and/or endogenous “danger” associated molecular patterns (DAMPs) [44]. Polarization towards a proinflammatory immune response may lead to immunopathology, to infectious, allergic, or autoimmune disease (Figure 2). An already infection-arouse immune system may be more reactive to subsequent exogenous and endogenous immune stimulation [44].

Therefore it is possible that in the described patient the* M. tuberculosis* infection due to stimulation of PRRs contributed to the severe pathology of hepatosplenic schistosomiasis* mansoni* and that both* M. tuberculosis* and schistosome infection might have contributed to the development of ALS. Interestingly, the increased levels of IL-17 observed in individuals with latent* M. tuberculosis* infection compared to patients with tuberculosis were suggested to have a protective effect against tuberculosis [45]. The complete Freund's adjuvant, that consists of heat killed* M. tuberculosis, *has for long time been known to enhance the immune response. Other potential environmental triggers like vitamin D deficiency [46–48], a hypothetical loss of intestinal helminths after his transfer from Ghana to Italy [31], a hypothetical decreased induction of oral immune tolerance due to a reduced amount oral antigens [49], a possible induction of IL-17 due to changes of the intestinal flora [50–52], might have promoted a proinflammatory immune response polarization, thus contributing to ALS development in the described patient. Increased IL-17 levels have been detected in the circulation and tissues of human and murine lupus [53]. It was demonstrated that IL-17 promotes B cell survival and differentiation into antibody producing cells. Therefore IL-17 is suspected to promote humoral immunity against self-antigens [53]. It is possible that increased IL-17 has contributed to the development of autoantibodies detected in the described patient.

## 4. Conclusion

Because ALS is not a disease of infancy, it is probable that contributing factors have to accumulate over time and/or have to combine with each other in order to overcome the various physiologic compensation-, and repair-mechanisms, and cause disease. Besides the genetic background, environmental factors such as infections, xenobiotic substances, changes in the gut microbiota and vitamin D deficiency, may contribute to shift the balance of the immune response from a protective to a more destructive one [54]. Autoimmune disease associations with ALS raise the possibility of shared genetic or environmental risk factors [55]. Recent progress in immunology suggests that in the described patient an increased IL-17 level may have been a common pathogenic feature of the different diseases. If we consider ALS as a common outcome of a multitude of different risk factors, so in the future we have to learn to recognize and differentiate the various combinations of the contributing factors or subcategories of the disease. This will be a precondition for a combined therapeutic approach from different fronts and for interventions of immune modulation without abrogation of the protective part of the immune response. The reported clinical case suggests that the analytic methods of immunology, for example, measuring of cytokines and chemokines, will have to be introduced into daily clinical practice in order to progress towards better understanding and towards an effective treatment and prevention of amyotrophic lateral sclerosis.

## Figures and Tables

**Table 1:** Laboratory data.

Analyte	Reference range	Month 1	Month 4	Month 8
Leukocyte count (×10^3^/*μ*L)	4.3–11.0	**1.30**	**1.50**	**1.70**
Lymphocytes (×10^3^/*μ*L)	1.0–3.7	**0.50**	**0.70**	**0.70**
CD4 T cells %	31–60	50		53
CD4 T cells (/*μ*L)	410–1590	**311**		**315**
Hemoglobin (g/dL)	12–16	12.9	13.4	13.8
Platelet count (×10^3^/*μ*L)	140–450	**36**	**33**	**51**
PT INR	<1.20	**1.49**	**1.44**	**1.22**
PTT RATIO	<1.20	**1.21**	1.15	1.05
CRP (mg/dL)	<0.50	**1.4**	0.01	0.03
*γ*-GT (U/L)	<60	**157**	**246**	**100**
AST (IU/liter)	<40	**56**	33	19
ALT (IU/liter)	<40	**59**	**87**	17
Gamma-globulin (%)	8–16.7	**19.6**	14.6	12.1
IgE (IU/mL)	<120	81	**175**	63
Vitamin B12 (pg/mL)	191–663		**1.057**	
Folic acid (ng/mL)	4.6–18.7		**3.89**	
25-OH-vitamin D (ng/mL)	31–100	**14**		
CPK (IU/liter)	40–230	**1,277**	**253**	**84**
ANA titer	<1 : 80	**1 : 320**	**1 : 160**	**1 : 320**
C3 (mg/dL)	79–152	**68**	**73**	**78**
C4 (mg/dL)	16–38	**12**	**11**	**12**
Lupus anticoagulant panel				
aPTT-low phospholipid	<1.15	0.85		0.66
DRVVT ratio	<1.10	**1.20**		**1.14**
Anticardiolipin-Ab		**positive**		
Schistosoma-Ab			**positive**	

HIV-Ab, HTLV-I/II-Ab, HAV-IgM, HBs-Ag, HCV-Ab, HEV-Ab, CMV-IgM, TPPA, *Toxoplasma*-Ab, EBV-DNA, *Plasmodium falciparum*-Ag, and emoscopy for plasmodia: all negative.

Values out of the reference range are in bold.

ALT: alanine transaminase; AST: aspartate transaminase; ANA: antinuclear antibodies; CPK: creatine phosphokinase; CRP. C-reactive protein; DRVVT: dilute viper venome time; *γ*-GT: *γ*-glutamyltransferase; PT: prothrombine time; HTLV-I/II: human T lymphotropic virus I/II; PTT: partial thromboplastine time; and TPPA: Treponema pallidum particle agglutination.
